# Mobile health applications in the management of hyperglycemia in pregnancy: a mini-review of current tools and future perspectives

**DOI:** 10.3389/fcdhc.2026.1761584

**Published:** 2026-03-02

**Authors:** Fanny Valsecchi, Annalisa Giancaterini, Erika Pedone, Amelia Caretto

**Affiliations:** 1 Vita-Salute San Raffaele University, Milan, Italy; 2 Endocrinology, Metabolic Disease and Nutrition Unit, Pio XI Hospital, Azienda Socio-Sanitaria Territoriale Brianza, Desio, Italy; 3 Diabetes Research Institute, IRCCS San Raffaele Scientific Institute, Milan, Italy

**Keywords:** mobile application, diabetes in pregnancy, digital health, gestational diabetes, mHealth, mobile health, pre-gestational diabetes, telemedicine

## Abstract

Hyperglycemia in pregnancy (HIP), encompassing gestational diabetes mellitus (GDM) and pre-gestational diabetes mellitus (PGDM), constitutes a growing clinical challenge, impacting approximately 23 million live births annually worldwide and conferring substantial maternal and fetal risks. This mini-review evaluates mobile health (mHealth) applications for HIP management, focusing on glycemic monitoring, nutritional interventions, physical activity promotion, insulin dose titration, and postpartum surveillance. Reviewed applications facilitate data collection from glucometers and continuous glucose monitoring systems, deliver graphical analytics, tailored recommendations, artificial intelligence-driven coaching, and secure remote data exchange with healthcare professionals, thereby increasing patient adherence, glycemic regulation, and perinatal outcomes, including reductions in HbA1c, neonatal birthweight, and caesarean section rates. Key benefits include enhanced patient empowerment, streamlined telemedicine, and psychosocial support, supported by trials demonstrating superior glycemic indices and reduced hyperglycemic excursions. Nonetheless, challenges persist, including heterogeneous clinical validation, socioeconomic-digital disparities, data security imperatives, and the absence of comprehensive integrated platforms. Future perspectives focus on developing digital systems that combine sensors, artificial intelligence, and online clinics. These systems aim to improve coordinated care for women with HIP, make treatment more effective, enhance user satisfaction, and help healthcare providers use resources efficiently.

## 1 Introduction

Diabetes in pregnancy is referred to any kind of hyperglycemia in pregnancy (HIP) and it includes gestational diabetes (GDM), in which hyperglycemia is diagnosed in pregnancy, and pre-gestational diabetes (PGDM) in which hyperglycemia is present before conception such as type 1 diabetes (T1DM), type 2 diabetes (T2DM) and other rare forms of diabetes (1, 2). Each of these situations increases the risk of maternal and fetal adverse outcomes and requires a tailored approach, but all require optimizing glucose control, monitoring glucose values, controlling gestational weight gain (GWG), and improving lifestyle. Moreover, intra-uterine exposure to hyperglycemia confers a higher risk of metabolic and cardiovascular diseases to offspring. According to the International Diabetes Federation (IDF), it is estimated that 23 millions of live births in 2024 faced some form of HIP. Of these, the majority (nearly 80%) were complicated by GDM, while 11% were complicated by PGDM and the remaining pregnancies were complicated by diabetes first diagnosed in pregnancy (3, 4).

Pregnant women with diabetes often use mobile devices to obtain information about their health status and to manage the care of their pregnancy. In this context, mobile Health (mHealth) may offer new opportunities for better management of HIP. mHealth is a component of eHealth. According to the Global Observatory for eHealth (GOe) of the World Health Organization (WHO), it is defined as “medical and public health practice supported by mobile devices” (5). The term mHealth most commonly refers to the use of devices, such as mobile phones, tablet computers, personal digital assistants, and wearable devices such as smart watches. It improves health services (disease prevention, health promotion, and management of medical conditions), gives information on clinical practice, and helps data collection. mHealth relies on the use of mobile applications (apps), such as those that use data from medical devices, apps to track wellness, and apps to manage and improve therapy adherence (6, 7). According to the 2020 ADA/EASD consensus report (6, 7), diabetes apps are divided into the following categories: dietary advice/monitoring, physical activity, glucose monitoring, insulin titration, and insulin delivery. When an app acts as a medical therapy, validated through clinical trials (RCTs) and approved by regulatory authorities, it is classified as digital therapeutics (DTx) (7). Not all mHealth software is DTx. Currently, there is not uniformity in the validation pathways of different mHealth apps. Some are fully reviewed and approved by regulatory authorities, while others are based on weak clinical evidence. This is why, according to the 2025 Standards of Care of the American Diabetes Association (8) there are insufficient data to provide recommendations for specific apps for diabetes management, education and support, in the absence of clinical trials and validation by regulatory bodies (9).

In this mini-review, we aim to summarize the main types of available mobile apps for pregnant women with HIP and to highlight benefits, limitations, and future perspectives for the use of mHealth applications in pregnancies complicated by diabetes.

## 2 Methods

An extensive search was conducted across PubMed/Medline, EMBASE, and ClinicalTrials.gov. In PubMed, the search strategy was developed using the string: [(mHealth) AND (pregnancy)] AND (diabetes), combining core concepts related to both gestational OR pregestational diabetes. A separate additional search was made on keywords such as “smartphone”, “web-based”, “wearable devices”, “telemedicine” AND “apps”. We then extended the search to include the postpartum period. We manually reviewed references to find further studies. We selected studies published from 2012 to 2025, with no language restrictions. Since this is a narrative review, we applied no journal or publication type limits to ensure a broad overview of recent evidence.

## 3 Potential application area of mHealth applications in pregnancies with diabetes

Diabetes in pregnancy requires a multidisciplinary approach in order to improve metabolic control, to guarantee a correct nutritional therapy, to encourage physical activity, and to monitor obstetrical issues. Mobile technology can help in different areas of HIP management. **Table 1** lists the characteristics of the main mobile applications and technologies designed for diabetes in pregnancy. Each mobile application for diabetes in pregnancy has at least one of the following features:

**Table 1:** Key features of leading applications for managing hyperglycemia during pregnancy.

Topic	Developers, year of study, app name or study name	Country	Population	Study design	Primary and secondary outcomes	Results
Acceptability and feasibility of mHealth interventions	Wickramasinghe N et al., 2016 and 2019, The Diamond Solution (61, 62)	Australia	10 women with GDM	Crossover clinical trial	Assess patient compliance and satisfaction, health professional satisfaction and level of glycemic control achieved.	The integration of the technological solution into standard care was favored over the provision of standard care alone.
	Harrison TN et al., 2017, The Virtual Visit for Women With Gestational Diabetes trial (63)	Denmark	10 women with GDM	Interventional study	Understand the acceptability and feasibility of the proposed intervention design.	The majority predicted they would be comfortable communicating with clinicians by telephone. One-half preferred telephonic virtual visits, one-half videoconferencing. Continuity of care was an important factor in facilitating confidence with the program.
	Jo S et al., 2016, GDM-Management App (18)	Korea	22 women with GDM	Qualitative study	The study was carried out in two phases: development of the app (analysis, design, development, and evaluation) and testing of user acceptability.	The application received high ratings for both perceived importance and usefulness; however, acceptability scores were comparatively low (after one week of use).
	Nørgaard SK et al., 2017, Pregnant with Diabetes App (11)	Denmark	139 pregnant women with T1DM and T2DM	Qualitative study	Assess use and knowledge of the app through structured questionnaire.	Pregnant women with pre-existing diabetes demonstrated a preference for receiving antenatal health information through a mobile application rather than via other media. Topics most frequently visited were “diet and carbohydrates”, “blood glucose” and “possible complications”.
	Miele et al., 2019, Trento Cartella Clinica del Cittadino Diabetes System (TreC-DS) (35)	Italy	10 pregnant women with T1DM	Qualitative study	Analyze processes, perceptions, interactions, and acceptability of TreC-DS.	Text-messaging system was well-received and useful, improving communication between patients and clinicians, supporting remote diabetes management, and reducing the need for face-to-face visits. Communication styles differed by patient group, with messages being more prescriptive for pregnant women.
	Khalil C et al., 2019, myDiabby App (27)	France	20 healthcare practitioners working in 20 different diabetes services and 15 women with GDM	Qualitative study	The aim is to identify factors of a broader adoption and diffusion of a telemonitoring solution.	Patients perceive telemonitoring systems as a convenient and effective method for managing their GDM, with healthcare professional endorsement supporting their use.
	Gance-Cleveland B et al., 2019, StartSmart App (19)	USA	Different cohorts of pregnant women and clinicians for each phase of development, including women with gestational diabetes and providers working with the target population	Qualitative study	Outcomes: acceptability, usability, feasibility, and the potential for integration of the app into care workflows.	Feasibility was confirmed by both patients and healthcare providers. Structuring screening, interventions, and referrals for multiple risk and protective factors may enhance adherence to prenatal care guidelines.
	Yee LM et al., 2020 and 2021, Sweet Mama App in the Texting for Diabetes Success (TDS) programme (64, 65)	USA	16 pregnant women with T2DM or GDM	Prospective qualitative study	Outcomes: app feasibility, acceptability, and areas for improvement.	The app contributed to reducing social isolation, increasing diabetes-related knowledge, improving patient comfort with the healthcare team, and reducing the logistical burdens of managing diabetes during pregnancy.
Gestational weight gain (GWG)	Yew TW et al., 2021, Habits-GDM, the SMART-GDM study (66)	Singapore	340 women with GDM	Randomized Controlled Trial (RCT)	Primary outcome: proportion of participants with excessive gestational weight gain (EGWG) in the intervention group vs the control group. Secondary outcomes: absolute GWG, glycemic control, and maternal, delivery, and neonatal outcomes.	Lower mean glucose levels and reduced glucose target exceedance in the intervention group. Regarded as composite, the overall neonatal complications were significantly lower in the intervention group. No significant differences in maternal weight gain, delivery mode, insulin use.
	Sandborg et al., 2021, Weight Management for Improved Pregnancy Outcomes (Healthy Moms), HealthyMoms App (67)	Sweden	305 pregnant women covering all BMI categories	RCT	Primary outcome: total GWG. Additional secondary outcomes: body fatness, dietary habits, moderate-to-vigorous physical activity (MVPA), glycemia, and insulin resistance.	No statistically significant effect on GWG (the effect differed by pre-pregnancy BMI with overweight and obese people gaining less weight in the intervention group). The intervention group had higher scores for the Swedish Healthy Eating Index at follow-up than the control group. No differences in body fatness, MVPA, glycemia, and insulin resistance between the two groups.
Physical activity and lifestyle modifications	Potzel AL et al., 2022, TRIANGLE App (68)	Germany	66 women with prior GDM	RCT	Primary outcome: proportion of women achieving ≥3 out of 5 Diabetes Prevention Program goals (physical activity ≥150 min/week, fiber intake ≥15 g/1,000 kcal, fat intake <30% of total energy intake, saturated fat intake <10% of total energy intake, and weight reduction ≥5%/weight maintenance. Secondary outcome: rate the TRIANGLE app using Mobile Application Rating Scale (uMARS).	No improvement for the intervention group modified intention-to-treat analysis. App’s quality and perceived impact: 4.3 ± 0.8/5 uMARS points.
	Khunti K et al., 2023; Highton PJ et al., 2025, Baby Steps programme (21, 22)	United Kingdom	293 women with prior GDM	RCT	Primary outcome: change in physical activity at 12 and 48 months. Secondary outcomes in 2023 study: self-efficacy for exercise, quality of life and anxiety and depression. Secondary outcomes in 2025 study: body weight, BMI, hip and waist circumferences, blood pressure, resting heart rate, HbA1c and lipid profile; questionnaires: self-efficacy for exercise, QoL, anxiety and depression at 48 months.	Between-group difference of additional 500 steps per day in 2023 and 1000 steps per day in 2025 (no significant difference). Lower self-reported home-based physical activity, significantly higher Self-Efficacy-for-Exercise and quality of life scores, lower anxiety score in the intervention group at 12 months. No significant effects were observed for anxiety, depression, quality of life, or HbA1c at 48 months.
	MyGDMoving	Italy	–	–	The aim is to illustrate benefits of appropriate physical activity during pregnancy. Includes interactive sessions for entry data and questionnaires related to physical activity during pregnancy (IPAQ - International Physical Activity Questionnaire).	–
Blood glucose (BG) goals and metrics monitoring	Mackillop L et al., 2014; Hirst JE et al., 2015; Hirst JE et al., 2016; Mackillop L et al., 2018, GDm-Health App, the Trial of Remote Evaluation and Treatment of Gestational Diabetes Mellitus (TREAT-GDM) (12, 26, 51, 69)	UK	203 women with GDM	RCT	Primary outcome: change in mean BG. Secondary outcomes: glycemic control metrics beyond mean BG, change in HbA1c, mean fasting, pre-meal, and post-meal BG, time to starting pharmacological treatment, maternal outcomes and neonatal outcomes. Patient satisfaction, cost analysis, protocol compliance.	No significant difference in the rate of change in BG levels. Less preterm birth and caesarean delivery in the intervention group. Other glycemic, maternal, and neonatal outcomes were comparable. Women in the intervention group recorded significantly more daily glucose readings and reported higher satisfaction. No significant difference in direct health care costs.
	Borgen I et al., 2017; Skar JB et al., 2018; Borgen I et al., 2019; Garnweidner-Holme L et al., 2020, Pregnant+ App (13, 14, 70, 71)	Norway	238 women with GDM. 158 women completed post-partum OGTT	RCT	Primary outcome: 2-hour BG level at the routine post-partum OGTT. Secondary outcomes: mode of delivery, induction of labor, Apgar score, birth weight, transfer to neonatal intensive care unit (NICU), breastfeeding practice.	Statistically non-significant difference in post-partum 2-hour glucose or most secondary outcomes (birth weight, breastfeeding, NICU transfer). In terms of diet, both groups improved over time, but the app did not show an additional benefit beyond usual care.
	Bromuri et al., 2016, The Personal Health System (PHS) (72)	Switzerland	24 women with GDM	RCT	Outcomes: glycemic control and BG metrics including frequency and volume of BG readings entered. Usability, performance and reliability of the system.	Acceptability in the intervention-group was high. Overall median glucose values were lower in the telemedicine-group. Four out of six daily blood glucose values were significantly better controlled compared to standard care.
	Peleg M et al., 2017; Rigla M et al., 2018, MobiGuide (20, 73)	Spain	20 women with GDM	Pilot study	Primary outcome: feasibility and acceptability of a mobile decision-support system for GDM. Secondary outcome: compliance of the patients with BG monitoring.	The feasibility of the system was confirmed, compliance of patients with blood glucose monitoring was higher than that observed in a historical group of 247 patients, similar in clinical characteristics. A user questionnaire indicated a high level of acceptability of the telemedicine platform.
	Miremberg et al., 2018, Digital Glucose Monitoring in Gestational Diabetes, Glucose Buddy App (36)	Israel	120 women with GDM	RCT	Primary outcome: compliance defined as the actual blood glucose measurements/instructed measurements X100. Secondary outcomes: diabetes-control parameters, pregnancy, and neonatal outcomes.	Higher level of compliance, lower mean blood glucose, lower rates of off-target measurements both fasting and 1-hour post-prandial, and a lower rate of pregnancies requiring insulin treatment in the intervention group.
	Yang P et al., 2018, WeChat platform (54)	China	57 + 50 women with GDM, 50 with normal glucose tolerance	Controlled clinical trial	Outcomes: fasting and 2-hour post-prandial BG level, maternal and fetal outcomes.	Fasting and 2-hour post-prandial BG were significantly lower and premature delivery was significantly less likely in the intervention group. Caesarean section was more likely in the intervention group. Pregnancy-induced hypertension had a higher incidence in GDM compared to controls with normal glucose tolerance.
	Guo H et al., 2019, Dnurse app (74)	China	124 women with GDM	RCT	Outcomes: general conditions, compliance in using the app, blood glucose, HbA1c, GWG, pregnancy and neonatal outcomes.	The intervention group demonstrated higher compliance, reduced use of outpatient services, lower HbA1c levels prior to delivery, fewer off-target fasting and 2-hour post-prandial glucose measurements, and reduced GWG.
	Zhou Y et al., 2022, continuing medical care (CMC) programme (37)	China	119 women with GDM receiving insulin therapy	RCT	Primary outcome: medication adherence assessed by the 5-item Medication Adherence Report Scale. Secondary outcomes: insulin injection technique (IIT), insulin requirement, prepartal and puerperal glycemic control, hypoglycemia, and pregnancy and neonatal outcomes.	Higher medication adherence, higher patient percentage with appropriate IIT, lesser pre-prandial insulin dose, higher patient proportion with both qualified prepartal and puerperal fasting and prepartal 2-hour post-prandial BG, lower puerperal HbA1c, fewer hypoglycemia, and lower NICU admission rate were observed in the intervention group. Cesarean delivery rate was higher among intervention cases. Qualified prepartal glycemic control was related to high medication adherence and proper IIT. NICU admission was associated with gestational hypertension, deficient medication adherence and premature rupture of fetal membrane.
Smartphone application with educational purpose	Tumminia A et al., 2019, MySweetGestation App (10)	Italy	–	–	Offers comprehensive educational support, ranging from prevention and risk factors for developing diabetes during pregnancy to treatment and post-gestational follow-up strategies. This includes information on nutritional and insulin therapy, recommended gestational weight gain, glucose and ketone monitoring, and post-partum management and follow-up.	–
Post-partum support	O’Reilly et al., 2019, Health-e mums App	Australia	26 women with prior GDM	Pilot study	Participants joined four focus groups during the pilot-testing phase. The focus group transcripts were analyzed thematically to evaluate the app functionality and user-experience.	Women were predominantly satisfied with the overall app design and its functionality. Participants identified a need for personalization as sub-themes within the app functionality theme.
	Lim K et al., 2021, the Smartphone App to Restore Optimal Weight (SPAROW) trial, nBuddy App (46)	Singapore	200 women with GDM	RCT	Primary outcome: achievement of optimal weight at 4 months post-partum. Secondary outcomes: absolute weight loss, serum metabolic markers, self-reported nutritional intake, health education, and quality of life.	No statistical significance in achieving optimal weight at 4 months post-delivery. Reported significantly reduced caloric intake, higher health-directed behavior scores and increased emotional distress scores in the intervention group.
Web-based interventions	Adolfosson A et al., 2012; Berg M et al., 2013; Linden K et al., 2017, MOtherhood and DIABetes–web support, MODIAB-web (25, 75, 76)	Sweden	7 pregnant women with T1DM	Qualitative study + consecutive explorative analysis	Assist shared decision-making, building on a therapeutic alliance between the mother and the healthcare providers, and on the women’s disease-related documentation. Outcome: effect of a web-based support program on general well-being and self-efficacy in relation to diabetes management.	Positive feedback with the MODIAB-web prototype, with growing consideration of women’s blood glucose levels and person-centeredness for T1DM patients. No significant difference between intervention group and controls in Well-being (W-BQ12), Diabetes empowerment/self-efficacy (SWE-DES) at 6 months postpartum and in the measured secondary psychosocial outcomes (sense of coherence, diabetes distress, fear of hypoglycemia). Trend toward lower HbA_1_c in the intervention group in late pregnancy, but after adjusting for baseline, the difference was not statistically significant.
	Larsen B et al., 2020, Fit for Two: Incorporating Wearable Trackers Into Clinical Care for Pregnant Women With Diabetes (77)	USA	17 pregnant women with T2DM	Pilot study	Outcomes: measure feasibility through recruitment, retention, and adherence data, and acceptability using consumer satisfaction questionnaires and follow-up interviews. Potential efficacy was explored by examining changes in daily steps over time.	Adherence in wearing the Fitbit was relatively high, median wear adherence of 90% of days. The intervention was generally well accepted. Mean daily steps increased from baseline to week 3 and then decreased through week 12 (intervention time 10–27 weeks of gestation).
	Kytö M et al., 2023, Usability Study of the Sensors and eMOM GDM Application (eMOMGDM) (32)	Finland	10 women with GDM	Mixed methods study	Outcomes: self-management of GDM with self-tracking of continuous blood glucose and lifestyle factors. Evaluation of user experience perspectives.	CGM was the most useful sensor compared to 3 other physical activity sensors: activity bracelet, hip-worn sensor and electrocardiography sensor. The body site of sensors is fundamental for quality tracking and patients’ preference. high acceptability for wearable sensors.

- Collection of glucose data: from glucometers or continuous glucose sensors, automatically or with manual data entry;
- Graphs and reports to be shared with the physician in order to facilitate communication between women and healthcare providers (HCPs);
- Analysis of data: integration of information from glucose data, diet, physical activity and others in order to give customized advice;
- Decision support system for therapies adjustment;
- Tailored alarms;
- Educational function: information about pregnancy management, therapies and diet, diabetes acute complications;
- Coaching: virtual coaching with chatbot or artificial intelligence (AI) algorithms, sometimes direct connections with HCPs;

### 3.1 Nutrition and lifestyle applications

Nutritional therapy is fundamental in both women with GDM and with pre-existing diabetes, to balance macronutrients intake and to control glucose values. Moreover, women with PGDM frequently use carbohydrate (CHO) counting to optimize insulin doses at meals. According to the 2020 ADA/EASD consensus report (6), apps for nutritional advice offer databases of CHO, fat, protein, and energy content useful to plan meals or to decide rapid-insulin dosage, with or without CHO counting. There are no specific apps for CHO counting in pregnant women with diabetes, so women are advised to use common apps for carbo-counting, some with implementation with an AI algorithm (i.e., Carbs and Cal, MySugr, MyFitness Pal, Foodily, GoCARB). Nutritional advice for a proper diet is often integrated into specific apps for diabetes in pregnancy [i.e., My Sweet Gestation (10), Pregnant with Diabetes (11), GDm-Health (12), Pregnant + (13)]. For example, use of The Pregnant+ App, which provides nutritional advice, allowed participants to improve their dietary score from baseline to 36 weeks of gestation. Results showed indeed no significant difference between the intervention (Pregnant+ app + usual care) and the control (usual care only) in the healthy dietary score (HDS-P+) (14). Nutrition information can also be obtained from generic apps for pregnant women without diabetes, paying attention to choose the ones that promote a balanced diet and can be set up with personalized goals.

Nutritional therapy and physical activity are fundamental to gaining the right amount of gestational weight, as gaining more weight than suggested by the Institute of Medicine Guidelines is associated with adverse maternal and infant outcomes (15). Physical activity in pregnancy, unless there are obstetrical contraindications, has positive effects. It is recommended that pregnant women with diabetes exercise regularly during pregnancy, focusing on moderate-intensity aerobic activity (1). In women with GDM, regular physical activity is associated with improved glucose control and reduced insulin therapy (1). Smartphone technology has been demonstrated to help improving adherence to physical activity (16). Apps can support women to obtain information on the importance of physical activity, examples of physical exercises, personalized parameters and a physical activity tracker. For example, MyGDMoving (17) is an app designed for pregnant women with diabetes that illustrates the benefits of appropriate physical activity during pregnancy, contains interactive sections that require users to enter data related to their condition and includes questionnaires related to physical activity during pregnancy (IPAQ - International Physical Activity Questionnaire). All information can be shared with the diabetes care team. GDM-Management App (18) allows the woman to enter GDM and lifestyle data, including food intake, physical activity, and weight data, to receive tailored suggestions aligned with clinical recommendations. The StartSmart (19) also generates evidence-based recommendations tailored to each woman’s risk and protective factors. Some apps have a direct connection with activity trackers. For example, Mobiguide (20) includes an accelerometer to detect the level of physical activity. Baby Steps (21), a recent randomized controlled trial (RCT), gives importance to interactive bite-sized information resources in addition to face-to-face sessions, promoting competition among participants, reviewing the daily step count through a direct connection between the app and the activity tracker, thereby letting the women be able to set goals and record information such as body weight. At 48 months, the intervention group showed a non-significant between-group difference of approximately 1000 additional steps/day, but a significant improvement in self-efficacy for exercise was observed at 12 months (22). Other apps were designed for the general population without diabetes, but can be adapted for women with HIP, such as Pears App (16), originally designed for overweight and obese pregnant women. In 2014 a study was published using the e-Mom Roc app (23). It was based on an electronic-intervention, including website and mobile phone components, named e-Moms of Rochester (e-Moms Roc). Pregnant women in the intervention arm used the weight gain tracker and the diet and physical activity goal-setting tools. The majority preferred the weight gain tracker, only 40% the diet and physical activity goal-setting tools. Even if it was conducted among non-diabetic women, the results highlight how diet goals are frequently difficult to achieve. One year later, another intervention involving the development of an mHealth app, the Eating4Two app (24), was published. It aimed to support women through a GWG calculator, providing general dietary information. Pregnant, not diabetic, women especially appreciated having unlimited access to nutritional information.

### 3.2 Glucose monitoring and insulin therapy applications

During pregnancy with diabetes, it is of paramount importance to follow regular antenatal visits and to make constant adjustments to nutritional therapy or to insulin therapy. Especially after the COVID-19 pandemic, remote sharing of glucose and other clinical data with HCPs has become increasingly common. Telehealth visits, when combined with in-person visits, may improve outcomes, especially in women with GDM (1). In this context, mobile technology can integrate glucose and other clinical data and transmit them to HCPs. Some apps can be used as personal diaries where women can keep information about glucose values, food intake, insulin dosages, weight changes [i.e., MODIAB-web (25), GDM-Management (18), GDm-health (12, 26), Pregnant + (13), myDiabby (27)]. Some glucometers can directly send via Bluetooth capillary blood glucose data to the app (i.e., One Touch Verio, Contour Next, Beurer GLU50evo) and store them in a dedicated cloud. Other apps allow the patient to enter glucose data manually (i.e., MySugr) and share them with HCPs. Apps for glucose data storage often includes the “add note function” that can improve the patient/doctor relationship, because patients could use this function to express emotion and even convey complex communicative intentions (28). Other apps include color range indicators, text messages, or other systems to detect out-of-range patterns between visits, empowering patients to self-manage their disease (29, 30).

Continuous Glucose Monitoring (CGM) should always be offered to women with T1DM in pregnancy and can also be considered in women with GDM or T2DM during pregnancy (1, 31). Glucose values from sensors are displayed in mobile applications, such as dedicated apps provided by sensor producers (i.e. Dexcom Clarity, Carelink) or apps designed for the downloading of different brands of sensors (i.e. Tidepool, GLOOKO). According to the 2020 ADA/EASD consensus report (6, 7), glucose monitoring apps track glucose data and graphically display glucose levels to assist the patient and the healthcare team in managing glucose control. CGM was proven to be the most useful sensor compared to other physical activity sensors (bracelet, hip-worn sensor, and electrocardiography sensor), especially to learn associations between glucose and nutritional intake (32).

International guidelines suggest that insulin should be used to manage T1DM in pregnancy and that it is preferred for the management of T2DM in pregnancy and GDM (1, 31). Insulin therapy requires frequent dose adjustments, especially during pregnancy when glucose needs changes rapidly from week to week. In this context, insulin titration apps can help persons with diabetes calculate basal, prandial, and correction insulin doses. Many insulin titration apps have been developed for T2DM and T1DM patients (i.e., Voluntis Insulia, WellDoc Blue Star, Diabeo, Hygieia D-Nav, Dario Health, Vitadio) and are associated with improvement in HbA1c and time in range (TIR). For example, Diabeo is an app, classified as DTx, provides support in calculating the dose of basal insulin and rapid-acting insulin for people with T2DM and T1DM. The use of the Diabeo system on a smartphone combined with biweekly teleconsultations was effective in reducing HbA1c by 0.91% over six months in a cohort of people with T1DM. Furthermore, when combined with standard care with in-person visits at the usual times, it led to a reduction in HbA1c of 0.67% (33). The app has also been proven effective in supporting patients with T2DM and physicians in the initiation of basal insulin. In fact, the group of patients with T2DM who used Diabeo at the start of basal insulin therapy were able to more easily achieve adequate fasting capillary blood glucose levels and higher insulin doses without severe hypoglycemia (34). In the setting of diabetes in pregnancy, some apps can support women in dosing calculation, even at meals with a bolus calculator. TreC-Diabetes System app (35) is an app designed for T1DM pregnant women from the Italian Trentino Region that guarantees constant connection between women and HCPs, also in rural areas. It includes a decision-making system supported through features such as a carbohydrate-counter and an insulin bolus calculator. GDm-Health mobile app (12, 26) was designed to facilitate transmission of glucose data to HCPs who receive an alert if values were out of range and could consequently send messages to modify insulin doses. Also, other apps such as GlucoseBuddy (36) or myDiabby (27) facilitate communication between women and physicians to receive recommendations on insulin adjustments. A Chinese experiment involved the figure of pharmacists which helped in the interactive platform on CMC app for the communication with patients, suggesting insulin dosage adjustments (with endocrinologists’ contribution) and giving recommendations on the management of hyperglycemia and hypoglycemia events (37). In the group of apps for insulin therapy, it is worth mentioning the insulin delivery apps of insulin pumps or smartMDI that collect and display data and provide a decision support system, and the apps of automated insulin delivery systems (AID) that connect insulin pump and glucose sensors through a specific algorithm (i.e., Medtronic 780G, CamAPS, Control IQ). These apps are often used by women with T1DM in pregnancy. It is important to customize these apps with appropriate glucose targets for pregnancy according to local and international guidelines, as the standard glucose targets are those of the Ambulatory Glucose Profile (AGP) for the non-pregnant population.

### 3.3 Applications for post-partum follow-up

Women with a history of GDM are at increased risk of developing T2DM and cardiovascular diseases later in life (38), and their offspring are prone to suffer from metabolic disorders (39). The risk of developing T2DM can be reduced by 58% with lifestyle modification (40), but it is usually difficult to give adequate support to women in the post-partum period, when follow-up oral glucose tolerance test should be performed (41, 42). It is also challenging to engage women in lifestyle modification because they need to take care of a newborn, face emotional distress, and have more family and work demands (43). As women after delivery rely on online resources to gather health information (44), mobile apps can be a valuable tool to engage women post-partum. The Health-e Mums app used an evidence-based intervention that provides health coaching materials, diabetes screening reminders, structured goals, personalized automated feedback on body weight, diet, and physical activity progress (45). The app was found to be functional and useful to engage in lifestyle behaviors change and regular diabetes screening. The Smartphone App to Restore Optimum Weight (SPAROW) trial was an RCT designed to investigate the efficacy of a smartphone app in restoring optimal weight following delivery in women with GDM (46). In the intervention group, women used the Nutritionist Buddy (nBuddy) app, recording data about weight, meals, and activity and having web-based interaction with a team of dieticians, physiotherapists, and occupational therapists. While controls received standard care with routine postnatal visits. The women in the intervention group reached an optimal weight more frequently at 4 months control and reported healthier behaviors and lower caloric intake (46).

## 4 Benefits of mHealth applications in pregnancies with diabetes

The use of mHealth apps in pregnancies with diabetes has been proven effective in improving clinical outcomes. According to the systematic review of Eberle and colleagues (47), mHealth apps contribute to lower HbA1c values, lower fasting and 2-hour after-meal blood glucose levels, and lower mean blood glucose levels. Moreover, they demonstrated a trend towards better patient compliance, lower neonatal birth weight, and lower rate to neonatal intensive care units, and they reported more vaginal deliveries in women who used apps. Better glucose control may also be derived from the diabetes self-management support associated with mHealth apps use (48) and the enhanced women’s compliance with glucose monitoring and treatment (49). The integration of digital nudging strategies and structured self-monitoring may enhance women’s awareness of their metabolic profile, lifestyle choices, and therapeutic management, particularly during the initial weeks post-diagnosis, which represent the most critical period due to the impact of transforming a joyful and intimate event into a highly medicalized pathway. This approach could effectively merge digital health with behavioral medicine.

Diabetes in pregnancy-specific apps can provide time - and cost- efficient tailored interventions for better management (50, 51), especially when apps have a high degree of personalization. Applications can be a valuable part of telemedicine and this system can streamline health interventions enabling better glucose control and lower risk of maternal and fetal complications in women with GDM (52). Women perceive apps as time-saving as they give the possibility to receive medical care from home (48). The lack of in-person interaction is not an issue as women attribute to mHealth apps a function of emotional support (48).

## 5 Limitations of mHealth applications in pregnancies with diabetes

Mobile phones are now widespread and constantly connected to the internet, allowing the use of complex apps. But it is necessary to consider that women with low income or deprivation may not have free access to the internet. Moreover, the health and digital literacy of women should be taken into account while developing mHealth apps. At present only a few app developers consider health literacy during the development phase or during the outcome evaluation phase (50).

Women complain about the lack of some important features in apps dedicated to the management of HIP. For example, the lack of enough didactic information, direct connections to glucometers, or direct communication with HCPs. In summary, the main limitation perceived by women is the lack of an “all-in-one” technology (48). In the future, AI will maybe contribute to designing more complete and personalized apps (53). In China, doctors have already tried to merge educational materials and clinical data (glucose values, blood pressure and weight) with the WeChat platform, a telemedicine system based on smartphones for women with gestational diabetes mellitus. This all-in-one system succeeded in improving glucose control (54).

Care in virtual modality is not always considered trustworthy as face-to-face care (48). Women need to be sure that they are receiving complete and updated care, also in virtual mode. Social influence, perceived system quality, and perceived information quality are fundamental to building a good trust in the app (55). A user-centered design approach in developing mHealth apps may help elaborate more trusted and friendly apps for the women. Also, involving HCPs in the developing phase may help increase the use and the impact of mHealth apps in clinical outcomes (48).

The safety and privacy of the data used in the app should be guaranteed, not only for the woman but also for the unborn child. In the USA, the Food and Drug Administration (FDA) strictly regulates medical devices with the “Digital Health Innovation Action Plan” (56). While in Europe there is the consortium “Digital Health Europe” to support the digital health transition (57). Regulatory gaps persist as many apps lack validation in accordance with the most recent ADA Standards.

Equity issues should be considered. People with low incomes may lack digital devices or internet connection and face literacy barriers. Moreover, the language of mHealth apps is usually English, other languages may be underrepresented, and this may limit the use in a multicultural context.

Regarding the studies included in this narrative review, many of the included papers are limited by small sample sizes as they report single-center experiences. This may lead to a reduction of the statistical power of the studies and to a lack of reproducibility or also increase the impact of confounding factors. Despite this, the results reported could serve as a basis for more structured research. Similarly, while the results of the few available RCTs differ in statistical significance, the trend towards the utility of implementing app-based tools is confirmed.

## 6 Future perspectives

**Figure 1:**
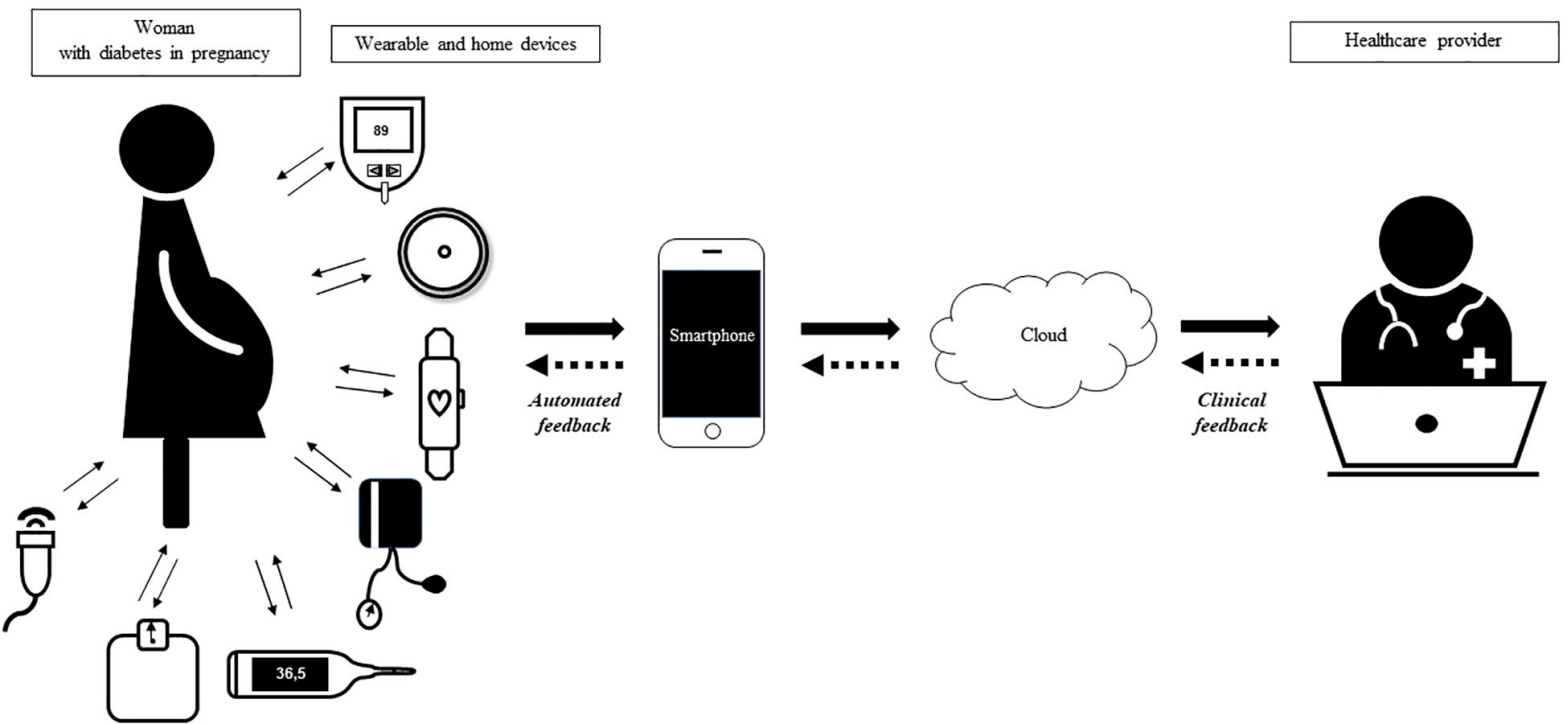
Digital ecosystem for pregnant woman with hyperglycemia in pregnancy. Woman is monitored with wearable and home devices (i.e. glucometer, glucose sensors, smartwatch, blood pressure cuffs, thermometer, weight scale, portable ultrasound). Data are transmitted to a mobile phone app and then to the cloud using a secure connection ensuring data encryption at rest and during transit, authentication and authorization for data display and data integrity. The healthcare provider analyzes data with an appropriate medical interface and provides personalized feedbacks (clinical feedback). Automated feedback derives from the smartphone/cloud directly to the patient, indicating real-time clinical alerts generated by integrated algorithms. Two-way arrows indicate the direction of information exchange and clinical or automated feedback.

A digital diabetes ecosystem is a system in which therapies, devices and digital tools are integrated to support people with diabetes in their daily life, reducing the burden of disease management (58). All these digital solutions are integrated in the digital diabetes virtual clinic (59). Diabetes in pregnancy can be part of the diabetes virtual clinic where pregnant women share data from glucose sensors and wearable devices with HCPs and receive support in pregnancy management (**Figure 1**). The digital ecosystem is open to integration with different kinds of devices. Glucose sensors and glucometers are necessary to detect glucose data and they can be integrated with other wearable devices such as activity trackers, portable blood pressure cuffs and pulse oximeters. Home digital devices can detect values about weight, body temperature and fetal wellbeing, with portable ultrasound or nonstress test devices. All data must be transmitted securely and in a standardized model (60). mHealth apps can serve as data loggers and diaries or as predictive models (with or without AI implementation), supporting diagnosis and management by HCPs who receive data with appropriate medical interface (60). Personal targets appropriate to pregnancy should be set and alarms can be helpful for women and HCPs to identify critical situations. As health systems are getting more and more digital, the virtual clinic can be implemented with data from electronic medical records and national registries. With this digital support, telemedicine can effectively support in-person visits. This ecosystem may help encounter the increasing number of pregnancies complicated by diabetes, the lack of HCPs and the increasing costs of health systems.

AI is at the moment underutilized in mHealth apps for HIP but may become a new frontier for future research in this field. The integration of AI within mHealth apps can support the predictive analysis of metabolic control and of pregnancy progress. Machine learning algorithms, for example, can serve for glucose forecasting, analyzing past glucose profiles and predicting future glucose levels. With risk stratification based on glucose trends, AI may estimate risk for future obstetrical or neonatal complications. Through machine learning algorithms, AI provides clinical decision support offering tailored therapeutic recommendations and real-time feedback. In the context of PGDM pregnancies, where the rapid increase in insulin requirements necessitates timely and precise management of therapy, AI support may become pivotal for quick insulin titration. Pregnancies, complicated by both GDM and PGDM, are characterized by an increase in insulin resistance as gestational weeks go by. AI can rapidly identify correlations between diet, physical activity, and blood glucose peaks that could otherwise be missed by manual analysis during gestational weeks. AI chatbots using natural language processing may give real-time answers to women’s questions and doubts, augmenting patients’ engagement.

## 7 Discussion

The mHealth applications are powerful tools in managing diabetes during pregnancy, as they enable better glycemic control, facilitate personalized care and enhance patient empowerment. Existing evidence suggests that applications built upon evidence-based content contribute significantly to improved pregnancy outcomes but efficacy is variable across studies. Systematic reviews like Eberle et al. (47) show consistent HbA1c reduction, but individual RCTs reveal discrepancies. For example, the UK RCT GDm-Health (12) found no significant mean blood glucose change but improved compliance and experimented fewer cesareans, contrasting the Glucose Buddy study of Miremberg et al. (36) which achieved lower off-target glucose and insulin needs. These inconsistencies arise from heterogeneous populations, small single-center populations and variable app integration (i.e., automated vs. manual data entry).

HIP includes both GDM and PGDM, which are different clinical scenarios. They are both characterized by the need to check and control hyperglycemia during gestation, but while GDM usually results in mild hyperglycemia and is limited in time, PGDM is a more complex disease with hyperglycemia lasting before conception and frequently leading to serious adverse obstetrical outcomes. mHealth apps dedicated to PGDM emphasize insulin titration support systems and CGM integration (i.e., TreC-Diabetes bolus calculator for type 1 diabetes, AID apps like CamAPS customized for pregnancy targets), addressing lifelong insulin needs conversely to GDM. GDM tools prioritize lifestyle (i.e., nutrition, physical activity) focusing on GWG and engagement for post -partum diabetes prevention. Consequently, outcomes may differ, studies on mHealth for PDGM highlight improvements in time in range and HbA1c (i.e., Diabeo DTx) while GDM trials focus on compliance and lifestyle and may lack on consistent neonatal or obstetrical benefits.

In this narrative review, we have summarized different global perspectives, illustrating how data-sharing apps between patients and HCPs are used even in small clinical experiences. Such technological communication tools enhance multidisciplinary care coordination and reduce accessibility barriers, ensuring that all pregnant women, regardless of language or socioeconomic status, can benefit from these digital health solutions.

Undoubtedly, there are still many areas of uncertainty that should be addressed by future research: i) there is the need for large multicenter RCTs powered enough for neonatal outcomes, ii) platforms should be combined with the automated integration of different devices and should be enhanced with AI, iii) trials should be addressed to low-literacy and rural settings, iv) cost-effectiveness should be more investigated, v) long-term effect of mHealth should be evaluated, possibly integrating with national registry in order to investigate the effect on T2DM and obesity prevention.

The use of apps and other digital tools in the management of HIP could represent a trend towards more organizational changes as reductions of in-person visits, thanks to timely medical feedback, combined with greater patient empowerment, thus reducing the burden of deferred decision-making. This constitutes a genuine clinical value rather than merely a technological one. While theoretical clinical trials are designed to be replicated globally, the integration with the real-world digital process will drive the large-scale implementation of “all-in-one” technology. It must be supported by active dissemination strategies to promote widespread adoption of the apps among public health professionals.

## References

[1] ElSayed NA McCoy RG Aleppo G Balapattabi K Beverly EA Briggs Early K . 15. Management of diabetes in pregnancy: standards of care in diabetes—2025. Diabetes Care. (2025) 48:S306–20. doi: 10.2337/dc25-S015, PMID: 39651985 PMC11635054

[2] Caretto A Scarascia Mugnozza F Valsecchi F Pedone E Pozzoni M Rosa S . Diabetes mellitus and pregnancy in Wolfram syndrome type 1: a case report with review of clinical and pathophysiological aspects. Front Med (Lausanne). (2025) 12:1656833. doi: 10.3389/fmed.2025.1639884, PMID: 40988749 PMC12450988

[3] Caretto A Pedone E Laurenzi A Bolamperti F Cellai C Pasi F . Type 1 diabetes diagnosed during pregnancy—an unusual but important challenge: a case series and review of literature. Front Med (Lausanne). (2025) 12:1656833. doi: 10.3389/fmed.2025.1656833, PMID: 41020242 PMC12464041

[4] Available online at: https://diabetesatlas.org/media/uploads/sites/3/2025/04/IDF_Atlas_11th_Edition_2025-1.pdf (Accessed December 4, 2025).

[5] Available online at: https://iris.who.int/server/api/core/bitstreams/ad1b13c0-7c82-47b4-8dd5-f0a26c3a3cc3/content (Accessed December 4, 2025).

[6] Fleming GA Petrie JR Bergenstal RM Holl RW Peters AL Heinemann L . Diabetes digital app technology: benefits, challenges, and recommendations. A consensus report by the european association for the study of diabetes (EASD) and the american diabetes association (ADA) diabetes technology working group. Diabetes Care. (2020) 43:250–60. doi: 10.2337/dci19-0062, PMID: 31806649

[7] Giancaterini A . Digital therapeutics: the innovation that will transform healthcare. J AMD. (2025) 28:37. doi: 10.36171/jamd25.28.1-2.6

[8] ElSayed NA McCoy RG Aleppo G Balapattabi K Beverly EA Briggs Early K . 7. Diabetes technology: standards of care in diabetes—2025. Diabetes Care. (2025) 48:S146–66. doi: 10.2337/dc25-er04b, PMID: 39651978 PMC11635043

[9] Caretto A . Digital Health: digital revolution in diabetology. J AMD. (2025) 28:22. doi: 10.36171/jamd25.28.1-2.5

[10] Tumminia A Vitacolonna E Sciacca L Dodesini AR Festa C Lencioni C . MySweetGestation”: A novel smartphone application for women with or at risk of diabetes during pregnancy. Diabetes Res Clin Pract. (2019) 158:107896. doi: 10.1016/j.diabres.2019.107896, PMID: 31669627

[11] Nørgaard SK Nichum VL Barfred C Juul HM Secher AL Ringholm L . Use of the smartphone application “Pregnant with Diabetes. Dan Med J. (2017) 64:A5417. 29115204

[12] Hirst JE Mackillop L Loerup L Kevat DA Bartlett K Gibson O . Acceptability and user satisfaction of a smartphone-based, interactive blood glucose management system in women with gestational diabetes mellitus. J Diabetes Sci Technol. (2015) 9:111–5. doi: 10.1177/1932296814556506, PMID: 25361643 PMC4495541

[13] Skar JB Garnweidner-Holme LM Lukasse M Terragni L . Women’s experiences with using a smartphone app (the Pregnant+ app) to manage gestational diabetes mellitus in a randomised controlled trial. Midwifery. (2018) 58:102–8. doi: 10.1016/j.midw.2017.12.021, PMID: 29329023

[14] Garnweidner-Holme L Henriksen L Torheim LE Lukasse M . Effect of the pregnant+ Smartphone app on the dietary behavior of women with gestational diabetes mellitus: secondary analysis of a randomized controlled trial. JMIR Mhealth Uhealth. (2020) 8:e18614. doi: 10.2196/18614, PMID: 33146620 PMC7673980

[15] Institute of Medicine (US) and National Research Council (US) Committee to Reexamine IOM Pregnancy Weight Guidelines . Weight Gain During Pregnancy. Washington, D.C: National Academies Press (2009).

[16] Kennelly MA Ainscough K Lindsay K Gibney E Mc Carthy M McAuliffe FM . Pregnancy, exercise and nutrition research study with smart phone app support (Pears): Study protocol of a randomized controlled trial. Contemp Clin Trials. (2016) 46:92–9. doi: 10.1016/j.cct.2015.11.018, PMID: 26625980

[17] Available online at: https://glucomenday.com/newplatform/it/app-software-for-diabetes-management/mygdmoving/ (Accessed December 4, 2025).

[18] Jo S Park HA . Development and evaluation of a smartphone application for managing gestational diabetes mellitus. Healthc Inform Res. (2016) 22:11. doi: 10.4258/hir.2016.22.1.11, PMID: 26893946 PMC4756053

[19] Gance-Cleveland B Leiferman J Aldrich H Nodine P Anderson J Nacht A . Using the technology acceptance model to develop startSmart: mHealth for screening, brief intervention, and referral for risk and protective factors in pregnancy. J Midwifery Womens Health. (2019) 64:630–40. doi: 10.1111/jmwh.13009, PMID: 31347784

[20] Peleg M Shahar Y Quaglini S Broens T Budasu R Fung N . Assessment of a personalized and distributed patient guidance system. Int J Med Inform. (2017) 101:108–30. doi: 10.1016/j.ijmedinf.2017.02.010, PMID: 28347441

[21] Khunti K Sukumar N Waheed G Gillies C Dallosso H Brough C . Structured group education programme and accompanying mHealth intervention to promote physical activity in women with a history of gestational diabetes: A randomised controlled trial. Diabetic Med. (2023) 40:108–30. doi: 10.1111/dme.15118, PMID: 37062022

[22] Highton PJ Funnell MP Tziannou A Rowlands AV Sukumar N Gillies CL . Structured group education programme and accompanying mHealth intervention to promote physical activity in women with a history of gestational diabetes (Baby Steps): 4-year follow-up of a randomised controlled trial. Diabetes Obes Metab. (2025) 28:754–8. doi: 10.1111/dom.70199, PMID: 41121954 PMC12673440

[23] Graham ML Uesugi KH Niederdeppe J Gay GK Olson CM . The Theory, Development, and Implementation of an e-Intervention to Prevent Excessive Gestational Weight Gain: e-Moms Roc. Telemedicine e-Health. (2014) 20:1135–42. doi: 10.1089/tmj.2013.0354, PMID: 25354350 PMC4270151

[24] Knight-Agarwal C Davis DL Williams L Davey R Cox R Clarke A . Development and pilot testing of the eating4two mobile phone app to monitor gestational weight gain. JMIR Mhealth Uhealth. (2015) 3:e44. doi: 10.2196/mhealth.4071, PMID: 26048313 PMC4526903

[25] Berg M Adolfsson A Ranerup A Sparud-Lundin for the U o, C . Person-centered web support to women with type 1 diabetes in pregnancy and early motherhood—The development process. Diabetes Technol Ther. (2013) 15:20–5. doi: 10.1089/dia.2012.0217, PMID: 23297670

[26] Mackillop L Loerup L Bartlett K Farmer A Gibson OJ Hirst JE . Development of a real-time smartphone solution for the management of women with or at high risk of gestational diabetes. J Diabetes Sci Technol. (2014) 8:1105–14. doi: 10.1177/1932296814542271, PMID: 25004915 PMC4455469

[27] Khalil C . Understanding the adoption and diffusion of a telemonitoring solution in gestational diabetes mellitus: qualitative study. JMIR Diabetes. (2019) 4:e13661. doi: 10.2196/13661, PMID: 31778118 PMC6913512

[28] Triberti S Bigi S Rossi MG Caretto A Laurenzi A Dozio N . The activeAgeing mobile app for diabetes self-management: first adherence data and analysis of patients’ in-app notes. (2018), 129–38. doi: 10.1007/978-3-030-01093-5_17, PMID: 41737702

[29] Grady M Katz LB Cameron H Levy BL . Diabetes app-related text messages from health care professionals in conjunction with a new wireless glucose meter with a color range indicator improves glycemic control in patients with type 1 and type 2 diabetes: randomized controlled trial. JMIR Diabetes. (2017) 2:e19. doi: 10.2196/diabetes.7454, PMID: 30291092 PMC6238868

[30] Grady M Cameron H Levy BL Katz LB . Remote health consultations supported by a diabetes management web application with a new glucose meter demonstrates improved glycemic control. J Diabetes Sci Technol. (2016) 10:737–43. doi: 10.1177/1932296815622646, PMID: 26685995 PMC5038536

[31] National Collaborating Centre for Women’s and Children’s Health (UK) . Diabetes in Pregnancy: Management of Diabetes and Its Complications from Preconception to the Postnatal Period. London: National Institute for Health and Care Excellence (UK) (2015). 25950069

[32] Kytö M Koivusalo S Tuomonen H Strömberg L Ruonala A Marttinen P . Supporting the management of gestational diabetes mellitus with comprehensive self-tracking: mixed methods study of wearable sensors. JMIR Diabetes. (2023) 8:e43979. doi: 10.2196/43979, PMID: 37906216 PMC10646680

[33] Charpentier G Benhamou PY Dardari D Clergeot A Franc S Schaepelynck-Belicar P . The diabeo software enabling individualized insulin dose adjustments combined with telemedicine support improves hbA1c in poorly controlled type 1 diabetic patients. Diabetes Care. (2011) 34:533–9. doi: 10.2337/dc10-1259, PMID: 21266648 PMC3041176

[34] Franc S Joubert M Daoudi A Fagour C Benhamou P Rodier M . Efficacy of two telemonitoring systems to improve glycemic control during basal insulin initiation in patients with type 2 diabetes: The TeleDiab-2 randomized controlled trial. Diabetes Obes Metab. (2019) 21:2327–32. doi: 10.1111/dom.13806, PMID: 31173451 PMC6771866

[35] Miele F Clementi S Gennaro R Nicolao I Romanelli T Speese K . Text messaging and type 1 diabetes management: qualitative study exploring interactions among patients and health care professionals. JMIR Diabetes. (2019) 4:e11343. doi: 10.2196/11343, PMID: 31094332 PMC6533872

[36] Miremberg H Ben-Ari T Betzer T Raphaeli H Gasnier R Barda G . The impact of a daily smartphone-based feedback system among women with gestational diabetes on compliance, glycemic control, satisfaction, and pregnancy outcome: a randomized controlled trial. Am J Obstet Gynecol. (2018) 218:453.e1–7. doi: 10.1016/j.ajog.2018.01.044, PMID: 29425836

[37] Zhuo Y Pan Y Lin K Yin G Wu Y Xu J . Effectiveness of clinical pharmacist-led smartphone application on medication adherence, insulin injection technique and glycemic control for women with gestational diabetes receiving multiple daily insulin injection: A randomized clinical trial. Prim Care Diabetes. (2022) 16:264–70. doi: 10.1016/j.pcd.2022.02.003, PMID: 35168915

[38] Bellamy L Casas JP Hingorani AD Williams D . Type 2 diabetes mellitus after gestational diabetes: a systematic review and meta-analysis. Lancet. (2009) 373:1773–9. doi: 10.1016/S0140-6736(09)60731-5, PMID: 19465232

[39] Vääräsmäki M Pouta A Elliot P Tapanainen P Sovio U Ruokonen A . Adolescent manifestations of metabolic syndrome among children born to women with gestational diabetes in a general-population birth cohort. Am J Epidemiol. (2009) 169:1209–15. doi: 10.1093/aje/kwp020, PMID: 19363101

[40] Ratner RE Christophi CA Metzger BE Dabelea D Bennett PH Pi-Sunyer X . Prevention of diabetes in women with a history of gestational diabetes: effects of metformin and lifestyle interventions. J Clin Endocrinol Metab. (2008) 93:4774–9. doi: 10.1210/jc.2008-0772, PMID: 18826999 PMC2626441

[41] Keely E . An opportunity not to be missed – how do we improve postpartum screening rates for women with gestational diabetes? Diabetes Metab Res Rev. (2012) 28:312–6. doi: 10.1002/dmrr.2274, PMID: 22228674

[42] Pennington AVR O’Reilly SL Young D Dunbar JA . Improving follow-up care for women with a history of gestational diabetes: perspectives of GPs and patients. Aust J Prim Health. (2017) 23:66–74. doi: 10.1071/PY15177, PMID: 28442034

[43] Nielsen KK Kapur A Damm P de Courten M Bygbjerg IC . From screening to postpartum follow-up – the determinants and barriers for gestational diabetes mellitus (GDM) services, a systematic review. BMC Pregnancy Childbirth. (2014) 14:41. doi: 10.1186/1471-2393-14-41, PMID: 24450389 PMC3901889

[44] Hearn L Miller M Lester L . Reaching perinatal women online: the Healthy You, Healthy Baby website and app. J Obes. (2014) 2014:573928. doi: 10.1155/2014/573928, PMID: 24872891 PMC4020447

[45] O’Reilly SL Laws R . Health-e mums: Evaluating a smartphone app design for diabetes prevention in women with previous gestational diabetes. Nutr Dietetics. (2019) 76:507–14. doi: 10.1111/1747-0080.12461, PMID: 30109762

[46] Lim K Chan SY Lim SL Tai BC Tsai C Wong SR . A smartphone app to restore optimal weight (SPAROW) in women with recent gestational diabetes mellitus: randomized controlled trial. JMIR Mhealth Uhealth. (2021) 9:e22147. doi: 10.2196/22147, PMID: 33724204 PMC8088857

[47] Eberle C Loehnert M Stichling S . Effectivness of specific mobile health applications (mHealth-apps) in gestational diabtetes mellitus: a systematic review. BMC Pregnancy Childbirth. (2021) 21:808. doi: 10.1186/s12884-021-04274-7, PMID: 34865645 PMC8645100

[48] Sushko K Menezes HT Wang QR Nerenberg K Fitzpatrick-Lewis D Sherifali D . Patient-reported benefits and limitations of mobile health technologies for diabetes in pregnancy: A scoping review. Can J Diabetes. (2023) 47:102–13. doi: 10.1016/j.jcjd.2022.08.001, PMID: 36182614

[49] Garg N Arunan SK Arora S Kaur K . Application of mobile technology for disease and treatment monitoring of gestational diabetes mellitus among pregnant women: A systematic review. J Diabetes Sci Technol. (2022) 16:491–7. doi: 10.1177/1932296820965577, PMID: 33118397 PMC8861802

[50] Chen Q Carbone ET . Functionality, implementation, impact, and the role of health literacy in mobile phone apps for gestational diabetes: scoping review. JMIR Diabetes. (2017) 2:e25. doi: 10.2196/diabetes.8045, PMID: 30291088 PMC6238859

[51] Mackillop L Hirst JE Bartlett KJ Birks JS Clifton L Farmer AJ . Comparing the efficacy of a mobile phone-based blood glucose management system with standard clinic care in women with gestational diabetes: randomized controlled trial. JMIR Mhealth Uhealth. (2018) 6:e71. doi: 10.2196/mhealth.9512, PMID: 29559428 PMC5883074

[52] Xie W Dai P Qin Y Wu M Yang B Yu X . Effectiveness of telemedicine for pregnant women with gestational diabetes mellitus: an updated meta-analysis of 32 randomized controlled trials with trial sequential analysis. BMC Pregnancy Childbirth. (2020) 20:198. doi: 10.1186/s12884-020-02892-1, PMID: 32252676 PMC7137255

[53] Giaxi P Vivilaki V Iliadou M Palaska E Diamanti A Gourounti K . The impact of mobile health (mHealth) apps on gestational diabetes: A systematic review. Cureus. (2025) 17:e79375. doi: 10.7759/cureus.79375, PMID: 39980710 PMC11841959

[54] Yang P Lo W He Z Xiao X . Medical nutrition treatment of women with gestational diabetes mellitus by a telemedicine system based on smartphones. J Obstetrics Gynaecology Res. (2018) 44:1228–34. doi: 10.1111/jog.13669, PMID: 29797375

[55] Mulyani EY Rakhmawati T Damayanti S Sumaedi S Yuda Bakti IGM . Pregnant women and mobile apps: Unraveling pregnant women’s adoption of mobile pregnancy education apps. Digit Health. (2025) 11:20552076251392648. doi: 10.1177/20552076251392648, PMID: 41229933 PMC12602969

[56] Available online at: https://www.fda.gov/media/106331/download (Accessed December 4, 2025).

[57] Available online at: https://digitalhealtheurope.eu/ (Accessed December 4, 2025).

[58] Kerr D Klonoff DC Bergenstal RM Choudhary P Ji L . A roadmap to an equitable digital diabetes ecosystem. Endocrine Practice. (2023) 29:179–84. doi: 10.1016/j.eprac.2022.12.016, PMID: 36584818

[59] Phillip M Bergenstal RM Close KL Danne T Garg SK Heinemann L . The digital/virtual diabetes clinic: the future is now—Recommendations from an international panel on diabetes digital technologies introduction. Diabetes Technol Ther. (2021) 23:146–54. doi: 10.1089/dia.2020.0375, PMID: 32905711 PMC8098767

[60] Murrin EM Saad AF Sullivan S Millo Y Miodovnik M . Innovations in diabetes management for pregnant women: artificial intelligence and the internet of medical things. Am J Perinatol. (2025) 42:1540–9. doi: 10.1055/a-2489-4462, PMID: 39592107

[61] Wickramasinghe N Gururajan R . Innovation practice using pervasive mobile technology solutions to improve population health management. J Healthcare Quality. (2016) 38:93–105. doi: 10.1097/JHQ.0000000000000033, PMID: 26918811

[62] Wickramasinghe N John B George J Vogel D . Achieving value-based care in chronic disease management: intervention study. JMIR Diabetes. (2019) 4:e10368. doi: 10.2196/10368, PMID: 31066699 PMC6524451

[63] Harrison TN Sacks DA Parry C Macias M Ling Grant DS Lawrence JM . Acceptability of virtual prenatal visits for women with gestational diabetes. Women’s Health Issues. (2017) 27:351–5. doi: 10.1016/j.whi.2016.12.009, PMID: 28153743

[64] Yee L Taylor S Young M Williams M Niznik C Simon M . Evaluation of a text messaging intervention to support self-management of diabetes during pregnancy among low-income, minority women: qualitative study. JMIR Diabetes. (2020) 5:e17794. doi: 10.2196/17794, PMID: 32773367 PMC7445621

[65] Yee LM Leziak K Jackson J Strohbach A Saber R Niznik CM . Patient and provider perspectives on a novel mobile health intervention for low-income pregnant women with gestational or type 2 diabetes mellitus. J Diabetes Sci Technol. (2021) 15:1121–33. doi: 10.1177/1932296820937347, PMID: 32627582 PMC8442184

[66] Yew TW Chi C Chan SY van Dam RM Whitton C Lim CS . A randomized controlled trial to evaluate the effects of a smartphone application–based lifestyle coaching program on gestational weight gain, glycemic control, and maternal and neonatal outcomes in women with gestational diabetes mellitus: the SMART-GDM study. Diabetes Care. (2021) 44:456–63. doi: 10.2337/dc20-1216, PMID: 33184151 PMC7818327

[67] Sandborg J Söderström E Henriksson P Bendtsen M Henström M Leppänen MH . Effectiveness of a smartphone app to promote healthy weight gain, diet, and physical activity during pregnancy (HealthyMoms): randomized controlled trial. JMIR Mhealth Uhealth. (2021) 9:e26091. doi: 10.2196/26091, PMID: 33704075 PMC7995071

[68] Potzel AL Gar C Banning F Sacco V Fritsche A Fritsche L . A novel smartphone app to change risk behaviors of women after gestational diabetes: A randomized controlled trial. PloS One. (2022) 17:e0267258. doi: 10.1371/journal.pone.0267258, PMID: 35476681 PMC9045614

[69] Hirst JE Loerup L Mackillop L Farmer A Kenworthy Y Bartlett K . Digital blood glucose monitoring could provide new objective assessments of blood glucose control in women with gestational diabetes. Diabetic Med. (2016) 33:1598–9. doi: 10.1111/dme.13035, PMID: 26606543

[70] Borgen I Garnweidner-Holme LM Jacobsen AF Bjerkan K Fayyad S Joranger P . Smartphone application for women with gestational diabetes mellitus: a study protocol for a multicentre randomised controlled trial. BMJ Open. (2017) 7:e013117. doi: 10.1136/bmjopen-2016-013117, PMID: 28348183 PMC5372027

[71] Borgen I Småstuen MC Jacobsen AF Garnweidner-Holme LM Fayyad S Noll J . Effect of the Pregnant+ smartphone application in women with gestational diabetes mellitus: a randomised controlled trial in Norway. BMJ Open. (2019) 9:e030884. doi: 10.1136/bmjopen-2019-030884, PMID: 31719080 PMC6858205

[72] Bromuri S Puricel S Schumann R Krampf J Ruiz J Schumacher M . An expert Personal Health System to monitor patients affected by Gestational Diabetes Mellitus: A feasibility study. J Ambient Intell Smart Environ. (2016) 8:219–37. doi: 10.3233/AIS-160365, PMID: 39743787

[73] Rigla M Martínez-Sarriegui I García-Sáez G Pons B Hernando ME . Gestational diabetes management using smart mobile telemedicine. J Diabetes Sci Technol. (2018) 12:260–4. doi: 10.1177/1932296817704442, PMID: 28420257 PMC5851209

[74] Guo H Zhang Y Li P Zhou P Chen LM Li SY . Evaluating the effects of mobile health intervention on weight management, glycemic control and pregnancy outcomes in patients with gestational diabetes mellitus. J Endocrinol Invest. (2019) 42:709–14. doi: 10.1007/s40618-018-0975-0, PMID: 30406378

[75] Adolfsson A Jansson . Prototype for Internet support of pregnant women and mothers with type 1 diabetes: focus group testing. Psychol Res Behav Manag. (2012) 97:97–103. doi: 10.2147/PRBM.S32799, PMID: 22915948 PMC3417839

[76] Linden K Berg M Adolfsson A Sparud-Lundin C . Well-being, diabetes management and breastfeeding in mothers with type 1 diabetes – An explorative analysis. Sexual Reprod Healthcare. (2018) 15:77–82. doi: 10.1016/j.srhc.2017.12.004, PMID: 29389505

[77] Larsen B Micucci S Hartman SJ Ramos G . Feasibility and acceptability of a counseling- and mHealth-based physical activity intervention for pregnant women with diabetes: the fit for two pilot study. JMIR Mhealth Uhealth. (2020) 8:e18915. doi: 10.2196/18915, PMID: 33084584 PMC7641781

